# Unmasking Schistosomiasis in the Appendix: A Rare Cause of Acute Appendicitis

**DOI:** 10.7759/cureus.93443

**Published:** 2025-09-28

**Authors:** Bader F Alshamlan, Munirah O Alfouzan, Abdulaziz F Alrubaiaan, Fady F Nakhla, Mohammad A Alghadhban

**Affiliations:** 1 General Surgery, Kuwait Institute for Medical Specializations (KIMS), Kuwait City, KWT; 2 General Surgery, Ministry of Health (MOH) Kuwait, Kuwait City, KWT

**Keywords:** appendicitis, intestinal schistosomiasis, parasitic infection, schistosomal appendicitis, tropical surgery

## Abstract

Schistosomiasis is a parasitic disease primarily found in tropical and subtropical regions, where access to safe drinking water is limited and hygiene is often lacking. Although the involvement of the vermiform appendix is very rare, we report a case of a 40-year-old adult presenting with acute right iliac fossa pain, who underwent laparoscopic appendectomy. Histopathology revealed *Schistosoma* ova in the wall of the appendix, confirming schistosomiasis. This case highlights the importance of considering parasitic causes of appendicitis and the need for further management, especially in tropical and subtropical regions where parasitic infections are endemic. Since a complete blood count (CBC) is a standard blood test, eosinophilia may be suggestive of a parasitic cause of appendicitis, particularly in conjunction with a patient's history.

## Introduction

Schistosomiasis, popularly known as bilharziasis, is a parasitic infection caused by blood flukes of the genus *Schistosoma*. The condition was first described in 1851 by Theodor Bilharz, after whom the disease is named [1]. Five distinct species of *Schistosoma* are recognized for infecting humans. Within these species, the intestinal forms are *S. mansoni*, endemic in Africa and South America; *S. japonicum*, found in East Asia; *S. mekongi*, common in Laos and Cambodia; and *S. intercalatum*, which occurs in West and Central Africa [2].

Schistosomiasis, caused by trematodes of the genus Schistosoma, commonly affects the urinary and gastrointestinal systems. Although it rarely causes appendicitis, it cannot be excluded. Appendicitis due to parasitic infections remains a neglected issue, although a significant number of appendicitis cases in endemic regions occur due to different parasitic infections. Thus, studies suggest that 2% to 4% of appendicitis cases in endemic regions might occur due to parasitic infection [3,4]. Appendiceal schistosomiasis is rare and remains a largely neglected cause of the condition [5]. The involvement of the vermiform appendix by schistosomiasis is found in 4.2% of cases [6].

## Case presentation

**Figure 1:**
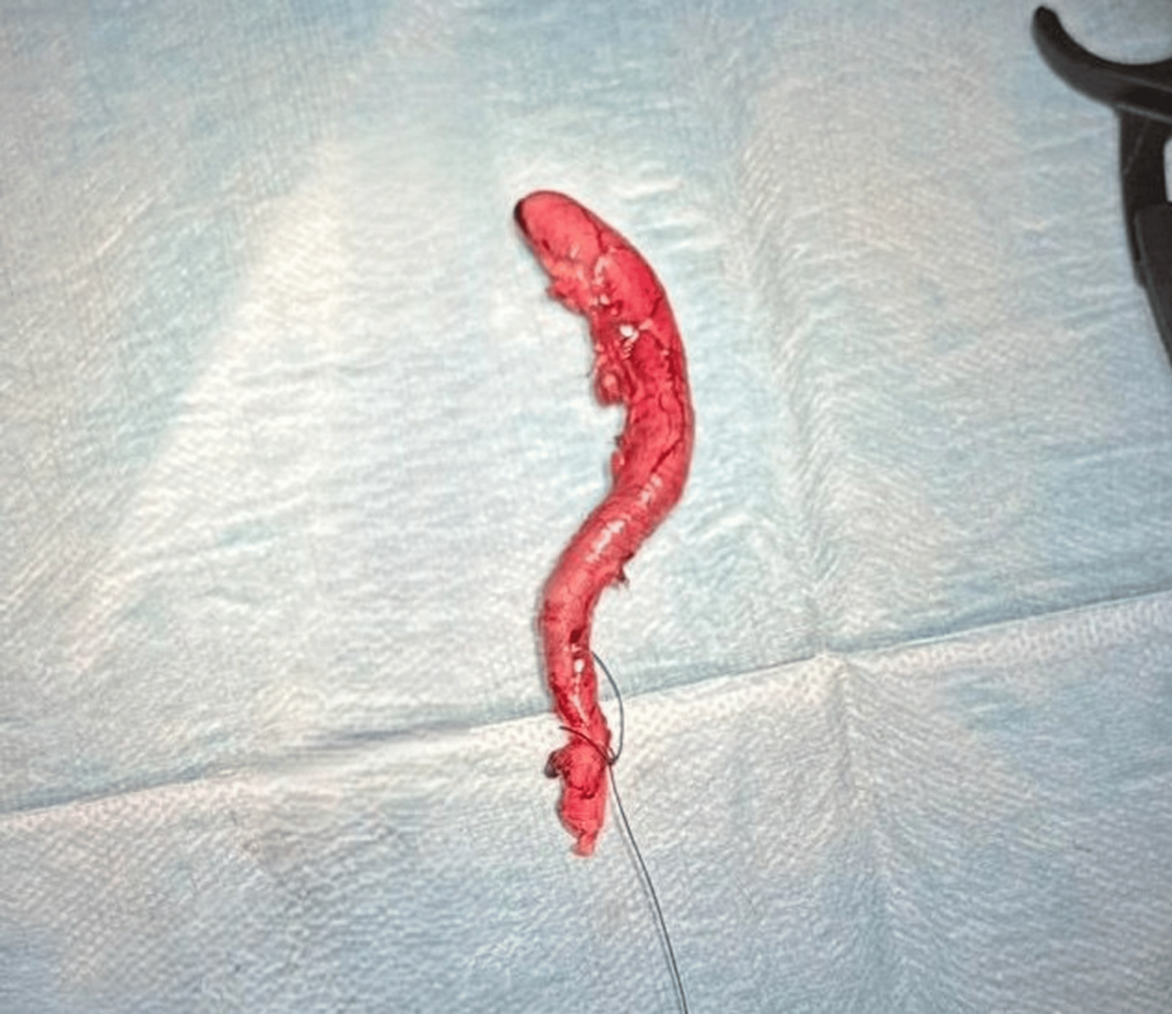
Gross specimen of resected appendix

A 40-year-old previously healthy Egyptian male from an endemic rural area presented with acute abdominal pain that initially started in the lower abdominal region and then became localized to the right iliac fossa, which lasted for one day, associated with nausea, anorexia, and low-grade fever. There was no history of hematuria or chronic diarrhea. The patient also had no history of allergies. On examination, the patient was febrile (38.2°C). Her other vitals were within normal range, and she had localized tenderness, rebound tenderness, and guarding in the right iliac fossa. Laboratory investigations revealed leukocytosis (WBC 15000); other labs were normal. Ultrasound of the abdomen suggested acute appendicitis along with a mild degree of fatty liver with a small hemangioma. A CT of the abdomen was not performed. The patient underwent a laparoscopic appendectomy. Intraoperative findings included an acutely inflamed appendix with a pyogenic membrane with a healthy base and pus in the pelvis (Figure 1).

**Figure 2:**
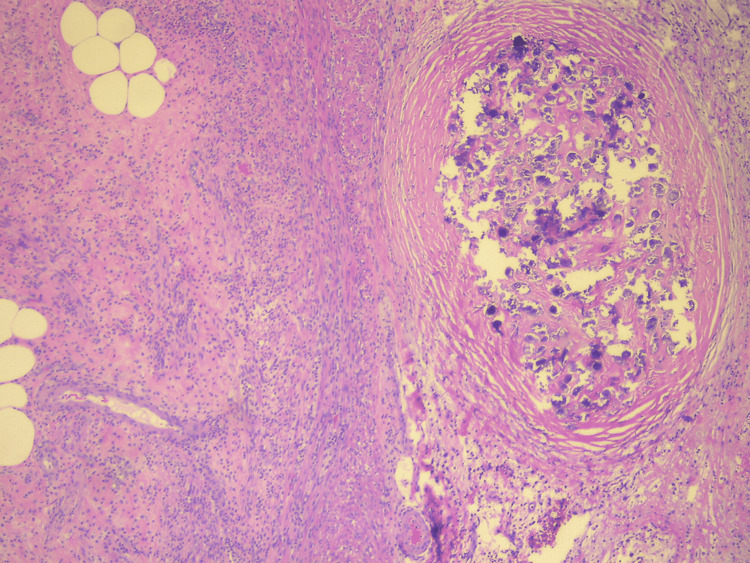
Section of appendix (wall) showing Schistosoma eggs with surrounding fibrosis (H&E stain 100X)

Postoperatively, the patient recovered uneventfully and was discharged on postoperative day one, remaining asymptomatic at the six-week follow-up. Histopathological examination revealed acute transmural inflammation with numerous calcified *Schistosoma* ova seen embedded in the appendicular wall, consistent with appendicular schistosomiasis (Figure 2).

## Discussion

Appendicular schistosomiasis, though rare should be considered a probable cause of acute appendicitis in endemic areas. However, it remains a neglected issue. The pathogenesis involves the deposition of Schistosoma ova in the appendix wall, leading to chronic granulomatous inflammation, fibrosis, and lumen obstruction, which predisposes the individual to acute appendicitis [7]. Diagnosis is rarely suspected preoperatively and is usually confirmed after histopathology. This is because, in many instances, patients do not exhibit significant symptoms, or the presentation of acute appendicitis masks these symptoms.

The pathophysiological mechanisms underlying schistosomal appendicitis remain incompletely understood. It has been proposed that the host immune response to schistosome egg deposition within the appendix plays a central role [8]. The resulting granulomatous inflammation not only targets the eggs but also damages the surrounding tissue, leading to either acute appendicitis or chronic inflammatory changes. Additionally, calcification of retained eggs may induce fibrosis and obstruction of the appendiceal lumen, thereby predisposing to secondary bacterial infection [9]. Management involves appendectomy to address acute inflammation, combined with medical treatment to eradicate the parasite and prevent recurrence. In endemic regions, parasitic causes should always be considered in cases of appendicitis, especially when eosinophilia is present.

This case highlights the importance of considering parasitic causes of appendicitis in patients residing in or belonging to endemic regions. Although in many cases diagnosis remains challenging due to vague symptoms, patient history might be suggestive, along with certain changes in CBC like eosinophilia [10]. Although histological examination of the appendix is standard in most nations, it is not the case in resource-limited settings, thus underscoring the importance of considering parasitic causes of appendicitis, its diagnosis, and the medical eradication of infections in patients in whom parasitic infections are suspected. Additionally, this case report shows that some patients might develop peritonitis, and in such cases, eradication of parasitic infection becomes more important and challenging [7]. Finally, it is also worth understanding that if the cause of appendicitis remains unidentified, the patient is quite likely to continue experiencing certain gastrointestinal symptoms post-appendicitis [11]. All these findings highlight the importance of considering parasitic infection in all patients living in endemic areas, keeping in mind that parasitic appendicitis is a possibility.

## Conclusions

Schistosomiasis of the appendix is a rare cause of acute appendicitis. A combination of surgical and medical management ensures complete treatment. Histopathology remains the gold standard for definitive diagnosis. In resource-limited settings, where histopathological examination is not standard, clinical diagnosis is vital, and careful history-taking, along with eosinophilia, might be suggestive of the issue. Appropriate antiparasitic therapy should be considered alongside the surgical management in such patients. Reporting such cases provides insight into the epidemiology and clinical presentation of schistosomal disease.

## References

[1] Rivadeneira DJ, Luo HS (2018). Intestinal schistosomiasis caused by Schistosoma japonicum: a literature review. J Infectiology & Epedemiol.

[2] Elbaz T, Esmat G (2013). Hepatic and intestinal schistosomiasis: review. J Adv Res.

[3] Zarbaliyev E, Celik S (2018). Parasitic appendicitis: a novel laparoscopic approach for the prevention of peritoneal contamination. Can J Infect Dis Med Microbiol.

[4] Altun E, Avci V, Azatcam M (2017). Parasitic infestation in appendicitis. A retrospective analysis of 660 patients and brief literature review. Saudi Med J.

[5] Limaiem F, Zaafouri M, Atallah A (2024). Hidden schistosomiasis unveiled by appendicular peritonitis: a case report. Int J Surg Case Rep.

[6] Badmos KB, Komolafe AO, Rotimi O (2006). Schistosomiasis presenting as acute appendicitis. East Afr Med J.

[7] Bello BM, Umar IA, Emetuma F (2023). Generalized peritonitis secondary to a ruptured schistosoma appendix: a case series. Niger J Gastroenterol Hepatol.

[8] Cox N, Yates P (2010). Schistosomiasis: a rare cause of acute appendicitis. J Surg Case Rep.

[9] Li JX, Fan HS (2023). Schistosomal appendicitis: a rare cause of a common surgical condition. J Surg Case Rep.

[10] Malta KK, Palazzi C, Neves VH, Aguiar Y, Silva TP, Melo RC (2022). Schistosomiasis mansoni-recruited eosinophils: an overview in the granuloma context. Microorganisms.

[11] Ahmed M (2023). Intestinal parasitic infections in 2023. Gastroenterology Res.

